# A new species of the genus *Rana* sensu lato Linnaeus, 1758 (Anura, Ranidae) from Wuyi Mountain, Fujian Province, China

**DOI:** 10.3897/zookeys.1065.67005

**Published:** 2021-10-26

**Authors:** Yanqing Wu, Shengchao Shi, Huiguang Zhang, Weicai Chen, Bin Cai, Van Chung Hoang, Jun Wu, Bin Wang

**Affiliations:** 1 Nanjing Institute of Environmental Sciences, Ministry of Ecology and Environment of China, Nanjing 210042, China Ministry of Ecology and Environment of China Nanjing China; 2 Chengdu Institute of Biology, Chinese Academy of Sciences, Chengdu 610041, China Chengdu Institute of Biology, Chinese Academy of Sciences Chengdu China; 3 Research and Monitoring Center, Wuyishan National Park, Wuyishan 354300, China Research and Monitoring Center, Wuyishan National Park Wuyishan China; 4 Key Laboratory of Beibu Gulf Environment Change and Resources Utilization of Ministry of Education, Nanning Normal University, Nanning 530001, China Nanning Normal University Nanning China; 5 Forest Resources and Environment Center, 300 Ngoc Hoi Road, Thanh Tri, Hanoi, Vietnam Forest Resources and Environment Center Hanoi Vietnam

**Keywords:** Molecular phylogenetic analyses, morphology, *Rana*, taxonomy

## Abstract

A new species of the frog genus *Rana* sensu lato from Wuyi Mountain, Fujian Province, China is described. Molecular phylogenetic analyses clustered the new species into the *R.johnsi* group and indicated that it was genetically divergent from its closely related species. The new species could be distinguished from its congeners by a combination of the following characters: body size medium, SVL 41.4–45.6 mm (42.9 ± 1.9 mm, n = 4) in adult males and 47.6–50.3 mm (n = 2) in adult females; adult male with a pair of internal subgular vocal sacs; lateroventral grooves present on tip of toes; webbing on fourth toes reaching the tip of toe; transverse skin ridges distinctly present on the dorsal surface of thigh and tibia, the number large (mean 26.5 ± 2.7, range 22–29, n = 6); breeding males possess creamy white nuptial pad with tiny velvety spines on the dorsal surface of the first finger, divided into three parts.

## Introduction

The brown frog genus *Rana* sensu lato Linnaeus, 1758 (Anura, Ranidae Batsch, 1796) is broadly distributed across Eurasia, Indochina, and North America (Frost 2021). The taxonomic arrangements in the group have been controversial for a long time (e.g., Fei et al. 1990; Dubois 1992; Fei et al. 2005, 2009, 2010, 2012; Frost et al. 2006; Che et al. 2007; Pyron and Wiens 2011; Jiang et al. 2020; Wang et al. 2020). A recent phylogenetic framework (Yuan et al. 2016) indicated that *Rana* sensu lato contained nine clades corresponding to eight subgenera and one unresolved species, i.e., *Rana*, *Amerana* Dubois, 1992, *Liuhurana* Fei, Ye, Jiang, Dubois & Ohler, 2010, *Aquarana* Dubois, 1992, *Lithobates* Fitzinger, 1843, *Zweifelia* Dubois, 1992, *Pantherana* Dubois, 1992, *Pseudorana* Fei, Ye & Huang, 1990, and *R.sylvatica* LeConte, 1825. Dubois et al. (2021) upgraded these subgenera to genera (except that the members of *Zweifelia* were placed in *Lithobates*) within *Ranites* Batsch, 1796, and established the new genus *Boreorana* Dubois, Ohler & Pryon, 2021 based on the type species *Ranasylvatica*. In the genus *Rana* sensu lato, 26 species have been recorded in China (Jiang et al. 2020; Wang et al. 2020; Frost 2021), which are *R.amurensis* Boulenger, 1886, *R.arvalis* Nilsson, 1842, *R.asiatica* Bedriaga, 1898, *R.chaochiaoensis* Liu, 1946, *R.chensinensis* David, 1875, *R.chevronta* Hu & Ye, 1978, *R.coreana* Okada, 1928, *R.culaiensis* Li, Lu, & Li, 2008, *R.dabieshanensis* Wang, Qian, Zhang, Guo, Pan, Wu, Wang, & Zhang, 2017, *R.dybowskii* Günther, 1876, *R.hanluica* Shen, Jiang, & Yang, 2007, *R.huanrenensis* Fei, Ye, & Huang, 1990, *R.jiemuxiensis* Yan, Jiang, Chen, Fang, Jin, Li, Wang, Murphy, Che, & Zhang, 2011, *R.jiulingensis* Wan, Lyu, & Wang, 2020, *R.johnsi* Smith, 1921, *R.kukunoris* Nikolskii, 1918, *R.longicrus* Stejneger, 1898, *R.luanchuanensis* Zhao & Yuan, 2017, *R.maoershanensis* Lu, Li, & Jiang, 2007, *R.omeimontis* Ye & Fei, 1993, *R.sangzhiensis* Shen, 1986, *R.sauteri* Boulenger, 1909, *R.shuchinae* Liu, 1950, *R.weiningensis* Liu, Hu, & Yang, 1962, *R.zhengi* Zhao, 1999, and *R.zhenhaiensis* Ye, Fei, & Matsui, 1995. Recent research on this genus discovered several new species from China (Yan et al. 2011; Yuan et al. 2016; Wang et al. 2017; Yang et al. 2017; Zhao et al. 2017), indicating that the diversity of the genus is probably underestimated.

Recently, in Wuyishan National Park, Wuyishan City, Fujian Province, China, we collected several specimens which can be assigned to *Rana* sensu lato based on morphology. Molecular phylogenetic analyses and detailed morphological comparisons indicated the specimens represented an undescribed species of the *R.johnsi* group. Herein we described it as a new species.

## Materials and methods

### Specimens

**Figure 1.**
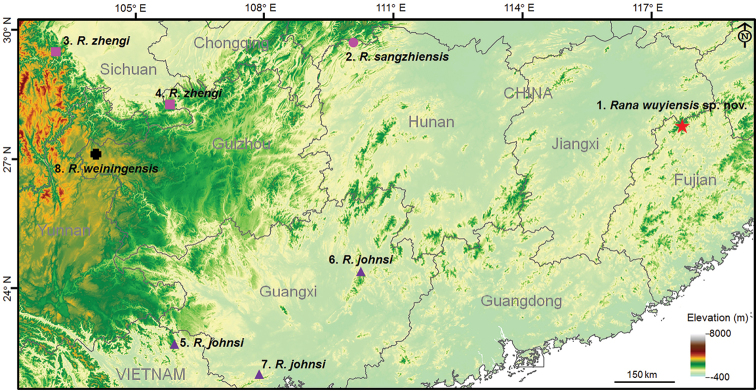
Locations for specimens used in this study. **1**. the type locality of *Ranawuyiensis* sp. nov., Wuyi Mountain, Fujian Province, China; **2**. the type locality of *R.sangzhiensis*, Sangzhi County, Hunan Province, China; **3**. the type locality of *R.zhengi*, Hongya County, Sichuan Province, China; **4**. another locality of *R.zhengi*, Gulin County, Sichuan Province, China; **5**. the locality for *R.johnsi* in Caobang Province, Vietnam; **6**. the locality for *R.johnsi* in Jinxiu County, Guangxi Province, China; **7**. the locality for *R.johnsi* in Shiwandashan Mountains, Guangxi Province, China; **8**. the type locality of *R.weiningensis*, Weining County, Guizhou Province, China.

Twelve unnamed specimens including four adult males, two adult females, and six tadpoles were collected from Wuyishan National Park, Fujian Province, China (Table 1, Fig. 1, Suppl. material 1). For comparisons, 39 specimens of the subgenus Rana were collected, i.e., six *R.zhengi* from Gulin County, Sichuan Province, China; five adult males, two adult females and six tadpoles of *R.sangzhiensis* from its type locality, Sangzhi County, Hunan Province, China; two adult males, one female, and one larval of *R.johnsi* from northern Vietnam; two larval of *R.johnsi* from Jinxiu County, Guangxi Province, China; eight adult males and six tadpoles of *R.johnsi* from Shiwandashan Mountains, Guangxi Province, China; and one adult male of *R.weiningensis* from its type locality, Weining City, Guizhou Province, China (Table 1, Fig. 1, Suppl. material 1). In the field, the frogs and tadpoles were euthanized using isoflurane, and the specimens were fixed in 75% ethanol. Muscle tissue samples were taken and preserved separately in 95% ethanol prior to fixation. The specimens were deposited in Chengdu Institute of Biology (**CIB**), Chinese Academy of Sciences, Nanning Normal University (**NNU**), and Institute of Ecology and Biological Resources (**IEBR**), Vietnam (for voucher numbers see Table 1 and Suppl. material 1).

**Table 1.** Information for samples used in molecular phylogenetic analyses in this study.

ID	Species	Voucher	Locality	16S	Cyt *b*	ND2	BDNF	RAG1	Tyr1
1	*Ranawuyiensis* sp. nov.	CIB WY20201106016	China: Fujiang: Wuyi Mountain	MZ337980	MZ355497	MZ355540	MZ355396	MZ355426	MZ355465
2	*Ranawuyiensis* sp. nov.	CIB WY20201106018	China: Fujiang: Wuyi Mountain	MZ337981	MZ355498	MZ355541	MZ355397	MZ355427	MZ355466
3	*Ranawuyiensis* sp. nov.	CIB WY20201106007	China: Fujiang: Wuyi Mountain	MZ337982	MZ355499	MZ355542	MZ355398	MZ355428	MZ355467
4	*Ranawuyiensis* sp. nov.	CIB WY20201106005	China: Fujiang: Wuyi Mountain	MZ337983	MZ355500	MZ355543	MZ355399	MZ355429	/
5	*Ranawuyiensis* sp. nov.	CIB WY20200913003	China: Fujiang: Wuyi Mountain	MZ337984	MZ355501	MZ355544	/	MZ355430	MZ355468
6	*Ranawuyiensis* sp. nov.	CIB WY20200913001	China: Fujiang: Wuyi Mountain	MZ337985	MZ355502	MZ355545	MZ355400	MZ355431	MZ355469
7	*Ranawuyiensis* sp. nov.	CIB WY20201106017	China: Fujiang: Wuyi Mountain	MZ337986	MZ355503	MZ355546	MZ355401	MZ355432	MZ355470
8	*Ranawuyiensis* sp. nov.	CIB WY20201106006	China: Fujiang: Wuyi Mountain	MZ337987	MZ355504	MZ355547	MZ355402	MZ355433	MZ355471
9	*Ranawuyiensis* sp. nov.	CIB WY20200913002	China: Fujiang: Wuyi Mountain	MZ337988	MZ355505	MZ355548	MZ355403	MZ355434	MZ355472
10	*Ranawuyiensis* sp. nov.	CIB WYS20200829003	China: Fujiang: Wuyi Mountain	MZ337989	MZ355506	MZ355549	MZ355404	MZ355435	MZ355473
11	*Ranawuyiensis* sp. nov.	CIB WYS20200829002	China: Fujiang: Wuyi Mountain	MZ337990	MZ355507	MZ355550	MZ355405	MZ355436	MZ355474
12	*Ranawuyiensis* sp. nov.	CIB WYS20200829001	China: Fujiang: Wuyi Mountain	MZ337991	MZ355508	MZ355551	MZ355406	MZ355437	MZ355475
13	*Ranazhengi*	CIB GL150097	China: Sichuan: Gulin County	MZ337992	MZ355509	MZ355552	MZ355407	MZ355438	MZ355476
14	*Ranazhengi*	CIB GL150091	China: Sichuan: Gulin County	MZ337993	MZ355510	MZ355553	MZ355408	MZ355439	MZ355477
15	*Ranazhengi*	CIB GL150088	China: Sichuan: Gulin County	MZ337994	MZ355511	MZ355554	MZ355409	MZ355440	MZ355478
16	*Ranazhengi*	CIB GL150011	China: Sichuan: Gulin County	MZ337995	MZ355512	MZ355555	MZ355410	MZ355441	MZ355479
17	*Ranazhengi*	CIB GL150010	China: Sichuan: Gulin County	MZ337996	MZ355513	MZ355556	MZ355411	MZ355442	MZ355480
18	*Ranazhengi*	CIB GL150068	China: Sichuan: Gulin County	MZ337997	MZ355514	MZ355557	MZ355412	MZ355443	MZ355481
19	*Ranasangzhiensis*	CIB SZ2012062103	China: Hunan: Sangzhi County: Tianping Mountain	MZ337998	/	/	/	/	/
20	*Ranasangzhiensis*	CIB SZ2012062106	China: Hunan: Sangzhi County: Tianping Mountain	MZ337999	/	/	/	/	/
21	*Ranasangzhiensis*	CIB SZ2012062104	China: Hunan: Sangzhi County: Tianping Mountain	MZ338000	MZ355515	/	MZ355413	MZ355444	MZ355482
22	*Ranasangzhiensis*	CIB TPS20190413-FTY1-5	China: Hunan: Sangzhi County: Tianping Mountain	MZ338001	MZ355516	MZ355558	MZ355414	MZ355445	MZ355483
23	*Ranasangzhiensis*	CIB TP20190413-36	China: Hunan: Sangzhi County: Tianping Mountain	MZ338002	MZ355517	MZ355559	MZ355415	MZ355446	MZ355484
24	*Ranasangzhiensis*	CIB TPS20190413-FTY1-1	China: Hunan: Sangzhi County: Tianping Mountain	MZ338003	MZ355518	MZ355560	MZ355416	MZ355447	MZ355485
25	*Ranasangzhiensis*	CIB TPS20190413-FTY1-2	China: Hunan: Sangzhi County: Tianping Mountain	MZ338004	MZ355519	MZ355561	MZ355417	MZ355448	MZ355486
26	*Ranasangzhiensis*	CIB TPS20190413-FTY1-3	China: Hunan: Sangzhi County: Tianping Mountain	MZ338005	MZ355520	MZ355562	MZ355418	MZ355449	MZ355487
27	*Ranasangzhiensis*	CIB TPS20190413-FTY1-4	China: Hunan: Sangzhi County: Tianping Mountain	MZ338006	MZ355521	MZ355563	MZ355419	MZ355450	MZ355488
28	*Ranazhengi*	SCUM 0405190CJ	China: Sichuan: Hongya County: Zhangcun	KX269206	/	/	/	/	/
29	*Ranasangzhiensis*	CIB SZ2012062102	China: Hunan: Sangzhi County: Tianping Mountain	MZ338007	MZ355522	/	MZ355420	MZ355451	MZ355489
30	*Ranajonhsi*	NNU 1910030	China: Guangxi: Shiwandashan Mountains	MZ338008	MZ355523	MZ355564	/	MZ355452	/
31	*Ranajonhsi*	NNU 1910023	China: Guangxi: Shiwandashan Mountains	MZ338009	MZ355524	MZ355565	/	MZ355453	/
32	*Ranajonhsi*	NNU 1910010	China: Guangxi: Shiwandashan Mountains	MZ338010	MZ355525	MZ355566	MZ355421	MZ355454	MZ355490
33	*Ranajonhsi*	NNU 1910032	China: Guangxi: Shiwandashan Mountains	MZ338011	MZ355526	MZ355567	/	MZ355455	/
34	*Ranajonhsi*	NNU 1910009	China: Guangxi: Shiwandashan Mountains	MZ338012	MZ355527	MZ355568	/	MZ355456	/
35	*Ranajonhsi*	NNU 1910001	China: Guangxi: Shiwandashan Mountains	MZ338013	MZ355528	MZ355569	/	MZ355457	MZ355491
36	*Ranajonhsi*	NNU 1910035	China: Guangxi: Shiwandashan Mountains	MZ338014	MZ355529	MZ355570	/	MZ355458	/
37	*Ranajonhsi*	NNU 1910021	China: Guangxi: Shiwandashan Mountains	MZ338015	MZ355530	MZ355571	/	MZ355459	/
38	*Ranajonhsi*	NNU 1910017	China: Guangxi: Shiwandashan Mountains	MZ338016	MZ355531	MZ355572	/	MZ355460	/
39	*Ranajonhsi*	NNU 1910002	China: Guangxi: Shiwandashan Mountains	MZ338017	MZ355532	MZ355573	/	MZ355461	MZ355492
40	*Ranajonhsi*	CIB 20070712-1	China: Guangxi: Jinxiu County	MZ338018	MZ355533	MZ355539	/	/	MZ355493
41	*Ranajonhsi*	CIB 20070712	China: Guangxi: Jinxiu County	MZ338019	MZ355534	/	MZ355422	/	/
42	*Ranajonhsi*	IEBR.A 4849	Vietnam: Cao Bang Province	MZ338020	MZ355535	MZ355574	MZ355423	MZ355462	MZ355494
43	*Ranajonhsi*	IEBR.A 4848	Vietnam: Cao Bang Province	MZ338021	MZ355536	MZ355575	MZ355424	MZ355463	MZ355495
44	*Ranajonhsi*	CIB 201204008	Vietnam: Cao Bang Province	MZ338022	MZ355537	MZ355577	/	/	/
45	*Ranaweiningensis*	CIB 20200806014	China: Guizhou: Weining County	MZ338023	MZ355538	MZ355576	MZ355425	MZ355464	MZ355496
46	*Ranaamurensis*	Tissue ID: MSUZP-SLK-RUS49	Russia: Tomskaya: Teguldetskii district	KX269203	KX269349	KX269418	/	/	/
47	*Ranaareolata*	KU 204370	USA: Kansas: Lyon: just S of Hartsford	AY779229	KX269300	KX269369	/	/	/
48	*Ranaarvalis*	Tissue ID: MSUZP-SLK-MKR21	Russia: Mordovia: Chamzinskii district	KX269197	KX269344	KX269413	/	/	/
49	*Ranaasiatica*	Tissue ID: KIZ-XJ0251	China: Xinjiang: 47tuan	KX269200	KX269346	KX269415	/	/	/
50	*Ranaaurora*	MVZ 188961	USA: California: Del Norte Co. Kings Valley	KX269212	/	KX269427	/	/	/
51	*Ranaberlandieri*	JSF 1136	USA: Texas: Hays: San Marcos	AY779235	KX269301	KX269370	/	/	/
52	*Ranablairi*	JSF 830	USA: Kansas: Douglas: Lawrence	AY779237	/	/	/	/	/
53	*Ranaboylii*	MVZ 148930	USA: California: Lake Co. along Butts Creek	KX269178	KX269299	KX269368	/	/	/
54	*Ranabwana*	QCAZ 13964	Ecuador: Loja: Río Alamor	AY779212	/	/	/	/	/
55	*Ranacapito*	TNHC 60195	USA: Florida: Marion: Archibold Biological Station	AY779231	/	/	/	/	/
56	*Ranacascadae*	TNHC-GDC 5297	no data	KX269176	KX269302	KX269371	/	/	/
57	*Ranacatesbeiana*	SCUM 0405176CJ	Pet trade	KX269208	KX269354	KX269423	/	/	/
58	*Ranachaochiaoensis*	SCUM 0405170CJ	China: Sichuan: Zhaojue	KX269192	KX269339	KX269408	/	/	/
59	*Ranachensinensis*	KIZ RD05SHX01	China: Shaanxi: Huxian	KX269186	KX269333	KX269402	/	/	/
60	*Ranachiricahuensis*	KU 194442	Mexico: Durango: Río Chico at Mexico Hwy	AY779225	KX269303	KX269372	/	/	/
61	*Ranaclamitans*	JSF 1118	USA: Missouri: Montgomery: 3 km W Danville	AY779204	KX269304	KX269373	/	/	/
62	*Ranacoreana*	MMS 223	South Korea	KX269202	KX269348	KX269417	/	/	/
63	*Ranaculaiensis*	KIZ SD080501	China: Shandong: Culaishan shan	KX269190	KX269337	KX269406	/	/	/
64	*Ranadabieshanensis*	AHU 2016R001	China: Anhui: Dabie Mountains	MF172963	/	/	/	/	/
65	*Ranadalmatina*	Tissue ID: MSUZP-NPUA-R-21-1	Ukraine: Zakarpatska: Uzhgorod District: Tschop	KX269198	/	/	/	/	/
66	*Ranadunni*	KU 194527	Mexico: Michoácan: Tintzuntzan: Lago Pátzcuaro	AY779222	KX269305	KX269374	/	/	/
67	*Ranadybowski*	Tissue ID: MSUZP-IVM-1d	Russia: Primorye region: Khasanskii District	KX269188	KX269335	KX269404	/	/	/
68	*Ranaforreri*	KU 194581	Mexico: Sinaloa: 37.9 km S	AY779233	GU184219	GU184250	/	/	/
69	*Ranagraeca*	ZMMU A-4293-1	Crna Gora (Montenegro): Niksic environs	KX269199	KX269345	KX269414	/	/	/
70	*Ranagrylio*	MVZ 175945	USA: Florida: Leon: Tall Timbers Research Station	AY779201	/	/	/	/	/
71	*Ranahanluica*	KIZ GX07112915	China: Guangxi: Maoershan shan	KX269191	KX269338	KX269407	/	/	/
72	*Ranaheckscheri*	MVZ 164908	USA: Florida: Gadsen-Leon	AY779205	/	/	/	/	/
73	*Ranahuanrensis*	MMS 231	South Korea	KX269183	KX269330	KX269400	/	/	/
74	*Ranaiberica*	ZMMU A-4292-1	Portugal: Porto: Valongo environs	KX269195	KX269342	KX269411	/	/	/
75	*Ranajaponica*	Tissue ID: KIZ-YPX11775	Japan: Isumi-shi: Chiba Prefecture	KX269220	KX269364	KX269434	/	/	/
76	*Ranajiemuxiensis*	KIZ HUN0708013	China: Hunan: Jiemuxi	KX269221	KX269365	/	/	/	/
77	*Ranajiulingensis*	SYS a005519	China: Jiangxi: Mt Guanshan	MT408985			/	/	/
78	*Ranajuliani*	TNHC 60324	Belize: Cayo District: Little Vaqueros Creek	AY779215	/	KX269375	/	/	/
79	*Ranakobai*	KUHE: 10051	Japan: Amami	AB685778	/	/	/	/	/
80	*Ranakukunoris*	KIZ CJ06102001	China: Qinghai: Qinghai Lake	KX269185	KX269332	KX269401	/	/	/
81	*Ranakunyuensis*	KIZ HUI040001	China: Shandong: Kunyu shan	KX269201	KX269347	KX269416	/	/	/
82	*Ranalatastei*	Veith 2003	Italy: Campagna: Seseglio 2 km	AY147946	AY147967	/	/	/	/
83	*Ranalongicrus*	NMNS 15022	China: Taiwan: Xiangtianhu: Miaosu	KX269189	KX269336	KX269405	/	/	/
84	*Ranaluteiventris*	MVZ 225749	USA: Washington: Pend Oreille Co. western	KX269213	KX269358	KX269428	/	/	/
85	*Ranamacrocnemis*	Tissue ID: MSUZP-LFM-12	Russia: Daghestan: Agulskiy District	KX269194	KX269341	KX269410	/	/	/
86	*Ranamacroglossa*	UTA A-17185	Guatemala: Sololá: Panajachel: Lake Atitlan	AY779243	KX269306	KX269376	/	/	/
87	*Ranamaculata*	KU 195258	Mexico: Oaxaca: Colonia Rodulfo Figueroa	AY779207	KX269307	KX269377	/	/	/
88	*Ranamagnaocularis*	KU 194592	Mexico: Sonora: Arroyo Hondo: 15.2 km N	AY779239	KX269308	KX269378	/	/	/
89	*Ranamontezumae*	KU 195251	Mexico: Morelos: Lagunas Zempoala	AY779223	KX269309	KX269379	/	/	/
90	*Ranamuscosa*	MVZ 149006	USA: California: Mono: Meadows western	AY779195	/	/	/	/	/
91	*Rananeovolcanica*	KU 194536	Mexico: Michoacan: Zurumbueno	AY779236	KX269310	KX269380	/	/	/
92	*Ranaokaloosae*	no voucher	USA: Florida: Santa Rosa: 5 km E	AY779203	/	/	/	/	/
93	*Ranaomeimontis*	SCUM 0405196CJ	China: Sichuan: Zhangcun: Hongya	KX269193	KX269340	KX269409	/	/	/
94	*Ranaomiltemana*	KU 195179	Mexico: Guerrero: Agua de Obispo Mexican Plateau	AY779238	KX269311	KX269381	/	/	/
95	*Ranaonca*	LVT 3542	USA: Nevada: Clark: Blue Point Spring Mexican	AY779249	/	/	/	/	/
96	*Ranaornativentris*	Tissue ID: KIZ-JP080101	Japan: Kyoto	KX269187	KX269334	KX269403	/	/	/
97	*Ranapalmipes*	AMNH A-118801	Venezuela: Amazonas: Río Mawarinuma	AY779211	/	/	/	/	/
98	*Ranapalustris*	ROM 21658	USA: New York: Middleburg eastern	KX269207	KX269353	KX269422	/	/	/
99	*Ranapipiens*	JSF 1119	USA: Ohio: Ottawa: Little Portage State Park	AY779221	/	/	/	/	/
100	*Ranapirica*	Tissue ID: MSUZP-NPFE-R-08-42	Russia: Sakhalinskaya Province: Makorovskiy District	KX269184	KX269331	/	/	/	/
101	*Ranapsilonota*	KU 195119	Mexico: Jalisco: 2.4 km NW Tapalpa	AY779217	KX269312	KX269382	/	/	/
102	*Ranapustulosa*	KU 200776	Mexico: Sinaloa: 2.1 km NE Santa Lucia	AY779220	KX269313	KX269383	/	/	/
103	*Ranapyrenaica*	ZFMK 65447-65448	Spain: Zuriza: Aragón	AY147950	AY147971	/	/	/	/
104	*Ranasakuraii*	Tissue ID: KIZJP080104	Japan: Tokyo	KX269205	KX269351	KX269420	/	/	/
105	*Ranasauteri*	SCUM 0405175CJ	China: Taiwan: Kaohsiung	KX269204	KX269350	KX269419	/	/	/
106	*Ranaseptentrionalis*	TNHC 72500	Canada: Ontario: Grey	KX269179	KX269314	KX269384	/	/	/
107	*Ranasevosa*	TNHC 60194	USA: Mississippi: Harrison	AY779230	/	/	/	/	/
108	*Ranashuchinae*	CIB HUI040009	China: Sichuan: Zhaojue	KX269210	KX269356	KX269425	/	/	/
109	*Ranasierrae*	MVZ 149007	USA: California: Mono Co. Meadows	KX269211	KX269357	KX269426	/	/	/
110	*Ranasierramadrensis*	KU 195181	Mexico: Guerrero: Agua de Obispo	AY779216	KX269315	KX269385	/	/	/
111	*Ranaspectabilis*	KU 195186	Mexico: Hidalgo: La Estanzuela	AY779227	KX269320	KX269390	/	/	/
112	*Ranasphenocephala*	JSF 845	USA: Kansas: Cherokee eastern	AY779251	KX269321	KX269391	/	/	/
113	*Ranasylvatica*	ID: MSUZP-SUNY-R-4-3	USA: New York: St. Lawrence Co.	KX269209	KX269355	KX269424	/	/	/
114	*Ranatagoi*	ID: MSUZP-NPJP-R-08-69	Japan: Kyoto	KX269214	KX269359	KX269429	/	/	/
115	*Ranatarahumarae*	KU 194596	Mexico: Sonora: 14.4 km E Yecora	AY779218	KX269322	KX269392	/	/	/
116	*Ranatemporaria*	ZMMU A-4288-1	Ukraine: Zakarpatska: Uzhgorod district	KX269196	KX269343	KX269412	/	/	/
117	*Ranatlaloci*	KU 194436	Mexico: Distrito Federal: Xochimilco	AY779234	KX269323	KX269393	/	/	/
118	*Ranatsushimensis*	NAP 4191	Japan: Nagasaki: Tsushima	KX269181	KX269329	KX269399	/	/	/
119	*Ranauenoi*	KIZ YPX36615	Japan: Nagasaki: Tsushima	KX269177	/	/	/	/	/
120	*Ranaulma*	OKW 135	Japan: Ryukyu Islands	KX269215	KX269360	KX269430	/	/	/
121	*Ranavaillanti*	KU 195299	Mexico: Oaxaca: 5.6 mi NE Tapanatepec	AY779214	/	KX269394	/	/	/
122	*Ranavibicaria*	TNHC GDC2266	Costa Rica: Cartago: El Emplame	KX269180	KX269324	KX269395	/	/	/
123	*Ranawarszewitschii*	JSF 1127	Panama	AY779209	KX269325	KX269396	/	/	/
124	*Ranayavapaiensis*	KU 194423	USA: Arizona: Greenlee: Apache National Forest	AY779240	KX269319	/	/	/	/
125	*Ranazhenhaiensis*	KIZ 803271	China: Zhejiang: Zhenhai	KX269218	JF939105	KX269433	/	/	/
126	*Odorranaversabilis*	HNNU A0019L	China: Hainan: Limu shan	KX269223	KX269367	KX269436	/	/	/
127	*Pelophylaxnigromaculatus*	SCUM 045199CJ	China: Sichuan: Hongya	KX269216	KX269361	KX269431	/	/	/
128	*Rugosatientaiensis*	SCUM 0405192CJ	China: Anhui: Huang shan region	KX269222	KX269366	KX269435	/	/	/
129	*Hylaranaguentheri*	SCUM H002CJ	China: Hainan: Sanya	KX269219	KX269363	/	/	/	/

### Molecular phylogenetic analyses

A total of 40 samples collected in this study was used in molecular analyses, encompassing twelve unnamed specimens from Wuyi Mountain, six *R.sangzhiensis*, six *R.zhengi*, 15 *R.johnsi*, and one *R.weiningensis* (Table 1). Total DNA was extracted using a standard phenol-chloroform extraction protocol (Sambrook et al. 1989). Three mitochondrial genes (16S rRNA, ND2, and Cyt *b*) and three nuclear DNA markers (Tyr, BDNF, and RAG1) were amplified and sequenced for the samples. Primer sequences used for PCR are presented in Table 2. Gene fragments were amplified under the following conditions: an initial denaturing step at 95 °C for 4 min; 36 cycles of denaturing at 95 °C for 30 s, 40 s at appropriate annealing temperature (Table 2); and extending at 72 °C for 70 s. PCR products were sequenced with primers same as used in PCR. Sequencing was conducted using an ABI3730 automated DNA sequencer. New sequences were deposited in GenBank (Table 1).

**Table 2.** Primers used for PCR and sequencing.

**Locus**	**Primer name**	**Sequences (5’ end 3’ end)**	**Temperature (°C)**	**Source**
16S	16SAR	AACGCTAAGATGAACCCTAAAAAGTTCT	55	Kocher et al. (1989)
	R16	ATAGTGGGGTATCTAATCCCAGTTTGTTTT	55	Sumida et al. (2000)
ND2	HERP322	TYCGARGACAGAGGTTTRAG	50	Yuan et al. (2016)
	HERP323	CAYCCACGRGCYATYGAA	51	Yuan et al. (2016)
Cyt *b*	HERP328	GAAAARCTRTCGTTGTWATTCAACTA	52	Yuan et al. (2016)
	HERP329	CTACKGGTTGTCCYCCRATTCATGT	53	Yuan et al. (2016)
Tyr	Tyr1G	TGCTGGGCRTCTCTCCARTCCCA	57	Bossuyt and Milinkovitch (2000)
	Tyr1B	AGGTCCTCYTRAGGAAGGAATG	57	Bossuyt and Milinkovitch (2000)
RAG1	AmpF2	ACNGGNMGICARATCTTYCARCC	50	Hoegg et al. (2004)
	AmpR2	GGTGYTTYAACACATCTTCCATYTCRTA	50	Hoegg et al. (2004)
BDNF	BDNF 2F	GAGTGGGTCAAGAGGAGG	55	Zhou et al. (2012)
	BDNF_2R	ACTGGGTAGTTCGGCATT	55	Zhou et al. (2012)

For phylogenetic analyses, the corresponding sequences for congeners especially for the topotypes of species in the subgenus Rana were downloaded from GenBank (Table 1), mainly derived from previous studies (Yuan et al. 2016; Wang et al. 2017; Wan et al. 2020). For phylogenetic analyses, corresponding sequences of one *Odorranaversabilis* (Liu & Hu, 1962) and one *Pelophylaxnigromaculatus* (Hallowell, 1861) were also downloaded (Table 1), and used as outgroups according to Yuan et al. (2016).

Sequences were assembled and aligned using the ClustalW module in BioEdit v.7.0.9.0 (Hall 1999) with default settings. The protein-coding gene (Cytb, ND2, BDNF, RAG1, and Tyr1) sequences were translated to amino acid sequences in MEGA v. 6.0 (Tamura et al. 2013), adjusted for open reading frames, and checked to ensure absence of premature stop codons. No-sequenced fragments were treated as missing data. For phylogenetic analyses based on mitochondrial DNA, the dataset was concatenated with mitochondrial gene sequences. The best partition scheme and the best evolutionary model for each partition were chosen for the phylogenetic analyses using PARTITIONFINDER v. 1.1.1 (Robert et al. 2012). In this analysis, 16S gene and each codon position of protein-coding mitochondrial gene were defined, and Bayesian Inference Criteria was used. As a result, the analysis suggested that the best partition scheme is 16S gene/each codon position of protein-coding gene, and selected GTR + G + I model as the best model for each partition. Phylogenetic analyses were conducted using maximum likelihood (ML) and Bayesian Inference (BI) methods, implemented in PhyML v. 3.0 (Guindon et al. 2010) and MrBayes v. 3.2 (Ronquist et al. 2012), respectively. For the ML tree, branch supports (bs) were drawn from 10,000 nonparametric bootstrap replicates. In BI, two runs each with four Markov chains were simultaneously run for 50 million generations with sampling every 1,000 generations. The first 25% trees were removed as the “burn-in” stage followed by calculations of Bayesian posterior probabilities (bpp), and the 50% majority-rule consensus of the post burn-in trees were sampled at stationarity. In addition, to access the genetic isolation between the undescribed species and its closely related species on nuclear DNA, one haplotype network for each nuclear gene dataset was constructed, using the maximum parsimony method in TCS v. 1.21 (Clement et al. 2000). Genetic distance of uncorrected-*p*-distance model on 16S gene sequences between the new species and its closely related species were estimated using MEGA.

### Morphological comparisons

All six adult specimens of the undescribed species were measured (Suppl. material 1). For comparisons, five adult male specimens of *R.sangzhiensis*, eleven adult male specimens of *R.johnsi*, and 22 adult male specimens of *R.zhengi* used in Jiang et al. (1997) were also measured (Suppl. material 1). The terminology and methods followed Fei et al. (2009). Measurements were taken with a dial caliper to 0.1 mm. Twenty-two morphometric characters of adult specimens were measured:

**ED** eye diameter (distance from the anterior corner to the posterior corner of the eye);

**FIIIL** third finger length (distance from base to tip of finger III);

**FIIL** second finger length (distance from base to tip of finger II);

**FIL** first finger length (distance from base to tip of finger I);

**FIVL** fourth finger length (distance from base to tip of finger IV);

**FL** foot length (distance from tarsus to the tip of fourth toe);

**HAL** hand length (distance from tip of third digit to proximal edge of inner palmar tubercle);

**HDL** head length (distance from the tip of the snout to the articulation of jaw);

**HDW** maximum head width (greatest width between the left and right articulations of jaw);

**IND** internasal distance (minimum distance between the inner margins of the external nares);

**IOD** interorbital distance (minimum distance between the inner edges of the upper eyelids);

**LAL** length of lower arm and hand (distance from the elbow to the distal end of the Finger IV);

**LW** lower arm width (maximum width of the lower arm);

**SL** snout length (distance from the tip of the snout to the anterior corner of the eye);

**SNT** distance between the nasal the posterior edge of the vent;

**SVL** snout-vent length (distance from the tip of the snout to the posterior edge of the vent);

**TFL** length of foot and tarsus (distance from the tibiotarsal articulation to the distal end of the Toe IV);

**THL** thigh length (distance from vent to knee);

**TL** tibia length (distance from knee to tarsus);

**TW** maximal tibia width;

**TYD** maximal tympanum diameter;

**UEW** upper eyelid width (greatest width of the upper eyelid margins measured perpendicular to the anterior-posterior axis).

To reduce the impact of allometry in adults, the correct value from the ratio of each character to SVL was calculated, and then was log-transformed for the following morphometric analyses. One-way ANOVA tests were conducted to test the significance of differences on morphometric characters between the undescribed species and its closely related species. The significance level was set at 0.05.

The morphological description follows the definition in Fei et al. (2009). Sex was determined by examining the gonads. The description of toe webbing followed Savage (1975). The description of digital pad followed Ohler (1995). Comparison characters of known congeners were obtained from the literature (Stejneger 1898; Liu 1946; Fei et al. 1990, 2005, 2009, 2012; Liu et al. 1993; Ye et al. 1993, 1995; Lu et al. 2007; Shen et al. 2007; Li et al. 2008; Yan et al. 2011; Wang et al. 2017; Zhao et al. 2017; Wan et al. 2020). We also examined a series of specimens of *Rana* (Suppl. material 1).

## Results

### Phylogenetic analyses

**Figure 2.**
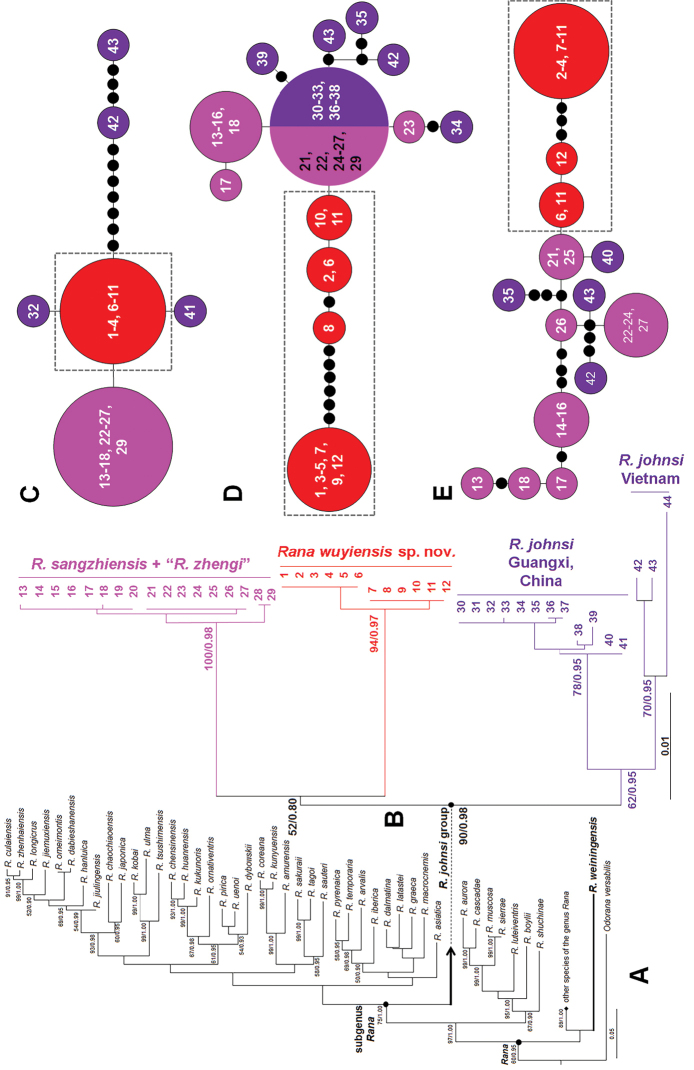
Phylogenetic relationships of *Ranawuyiensis* sp. nov. and its relatives **A** maximum likelihood (ML) tree reconstructed based on the 16S, ND2 and Cyt *b* gene sequences **B** a part of the tree highlighting the relationships of the *R.johnsi* group. ML bootstrap supports/Bayesian posterior probability was denoted beside each node. Sample 1–44 refer to Table 1**C–E** haplotype networks constructed based on sequences of nuclear genes BDNF, RAG1, and Tyr, respectively. Different species of the *R.johnsi* group were denoted as different colors.

ML and BI trees of the mitochondrial DNA dataset presented almost consistent topology (Fig. 2A, B). In mitochondrial DNA trees, all samples of the undescribed species were strongly nested into one clade (all supports = 100; Fig. 2B). The *R.johnsi* group was strongly supported as a monophyletic group containing all samples of *R.johnsi*, *R.sangzhiensis*, *R.zhengi*, and the undescribed species (all supports = 100; Fig. 2B). The *R.johnsi* group was clustered into the clade corresponding to the subgenus Rana (Fig. 2A). The *R.johnsi* group contained two clades. In the first clade, samples of *R.sangzhiensis* and *R.zhengi* were nested into a clade (all supports = 100), which was weakly clustered as the sister of the undescribed species clade (bs = 52; bpp = 0.80; Fig. 2B). In the second clade, three *R.johnsi* samples from Vietnam were clustered into one clade, which was sister to the clade containing samples of *R.johnsi* from two localities of Guangxi Province, China (Figs 1, 2B; Table 1). In addition, the topotype specimen of *R.weiningensis* was phylogenetically far from the *R.johnsi* group, and clustered as the basal clade of the genus *Rana* (Fig. 2A). Haplotype networks based on three nuclear genes all indicated that the undescribed species did not share haplotype with its closely related species *R.johnsi*, *R.sangzhiensis*, and *R.zhengi* (Fig. 2C–E), further indicating the genetic divergence between the undescribed species and its closely related species. As note, on each gene, samples of *R.sangzhiensis* and *R.zhengi* massively shared common haplotypes (Fig. 2C–E), indicating their very shallow genetic divergence. The genetic distance on 16S between all samples of undescribed species is less than 0.2% (range 0.0%-0.2%). The genetic distance between the species and its closely related species were as following: vs. *R.johnsi* from Vietnam 1.3% (range 1.1%–1.7%), vs. *R.johnsi* from Guangxi, China 0.8% (range 0.8%–0.9%), vs. *R.zhengi* 1.0% (range 0.9%-1.1%), and vs. *R.sangzhiensis* 0.9% (range 0.8%–1.1%), being similar to that between the latter four groups (range 0.8%–1.4%). As note, the genetic distance between *R.zhengi* and *R.sangzhiensis* was 0.2% (range 0.0%–0.4%), and that between *R.johnsi* from Vietnam and *R.johnsi* from Guangxi, China was 0.5% (range 0.4%–1.1%).

### Morphological comparisons

The *R.johnsi* group is phylogenetically clustered into the subgenus Rana, but this group could be identified from other species of the subgenus Rana by the tip of toes with lateroventral grooves (vs. absent in other species of subgenus Rana). The undescribed species could be assigned to this species group by a series of morphological characters: tip of toes flat with lateroventral grooves; body size medium, SVL 41.4–45.6 mm (42.9 ± 1.9 mm, n = 4) in adult males and 47.6–50.3 mm (n = 2) mm in adult females; dorsolateral fold distinct and thin, extending straight from posterior margin of upper eyelid to above groin; tympanum distinct, oval; tibio-tarsal articulation reaching forward beyond tip of snout when leg starched forward; skin ridges distinctly arranged on the dorsal surface of thighs and tibias; adult males with a pair of internal subgular vocal sacs; breeding males possess creamy white nuptial pad with tiny hoar velvety spines on the dorsal surface of the first finger, divided into three parts.

**Figure 5.**
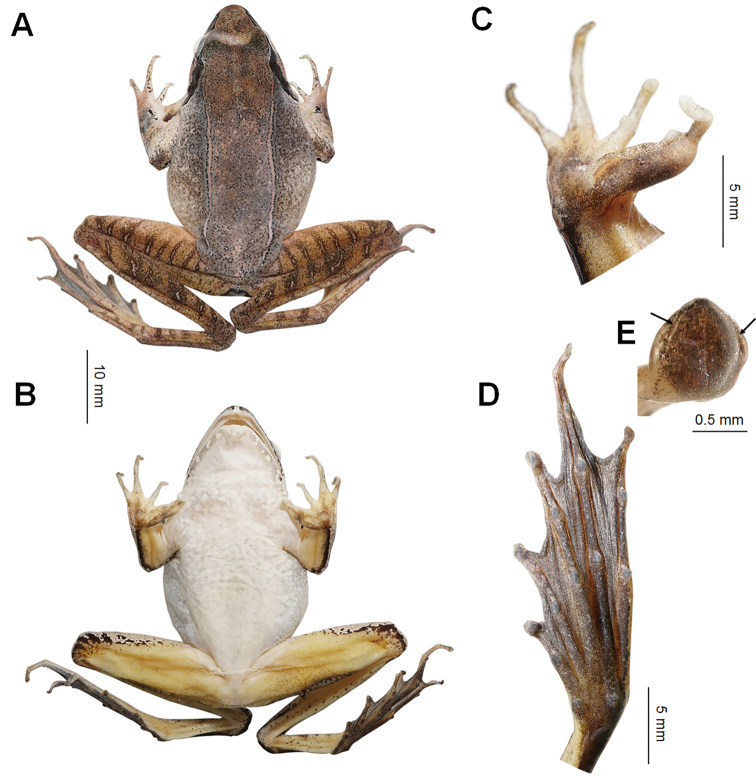
Photos of the holotype specimen CIB WY20200913003 of *Ranawuyiensis* sp. nov. **A** dorsal view **B** ventral view **C** ventral view of hand **D** ventral view of foot **E** ventral view of the toe highlighting the lateroventral grooves (arrows).

Although the *R.johnsi* group and *R.weiningensis* both have lateroventral grooves on the tip of toes, the undescribed species in the *R.johnsi* group could be easily distinguished from *R.weiningensis* by the following characters: males with internal subgular vocal sacs (vs. absent in the latter); males with lager body size (41.4–45.6 mm, n = 4 vs. 32.8–37.4 mm, n = 3 in the latter); and more developed webbing between toes (webbing on fourth toes reaching tip of toe vs. reaching distal subarticular tubercle in the latter).

**Figure 3.**
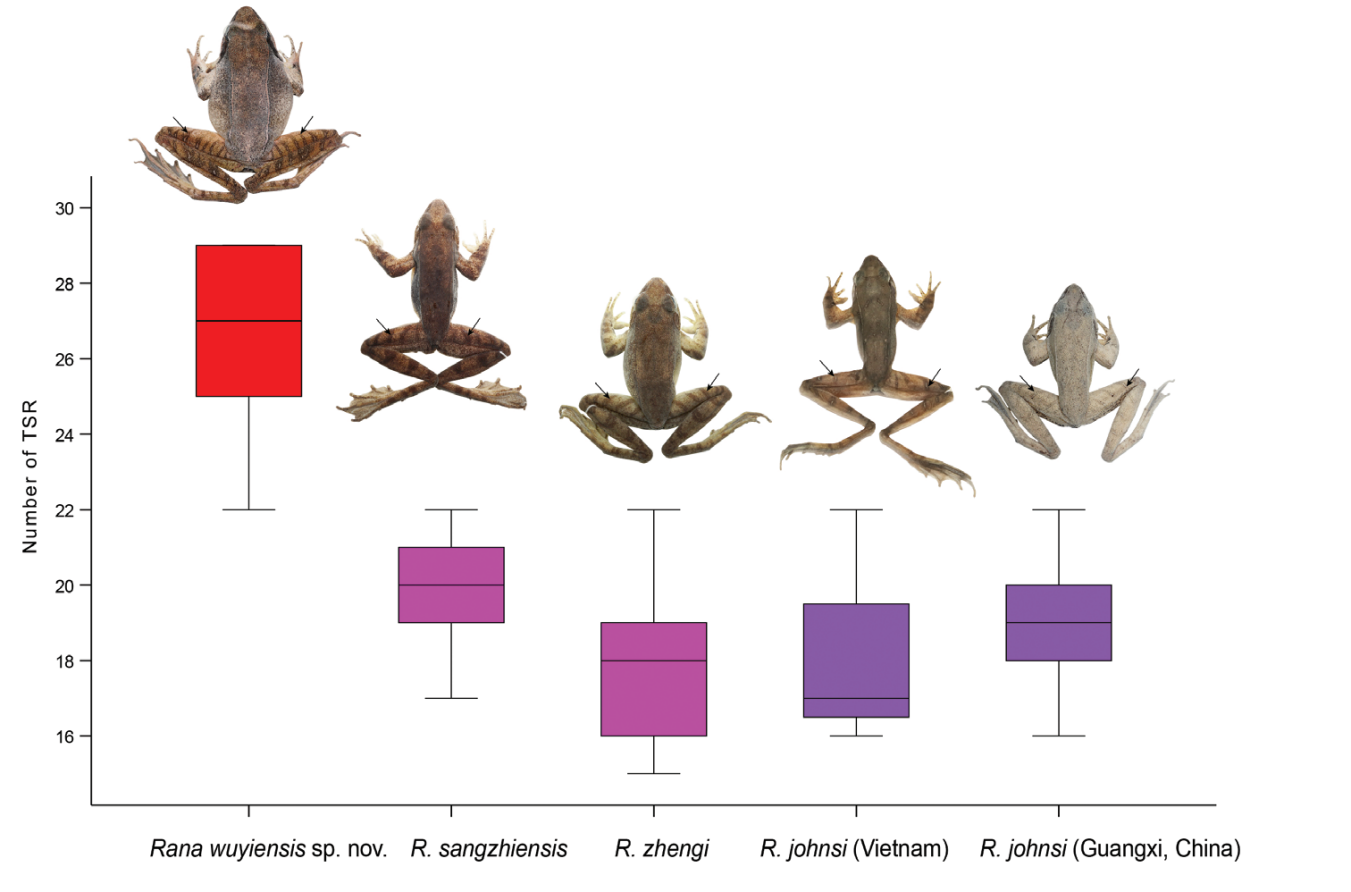
Box-plot showing the difference on the number of transverse skin ridges on the dorsal surface of thighs and tibias between different species. Specimens of different species: the holotype CIB WY20200913003 of *Ranawuyiensis* sp. nov., the topotype specimen CIB SZ2012061203 of *R.sangzhiensis*, the topotype specimen CIB 950300 of *R.zhengi*, the specimen NNU 1910009 of *R.johnsi* from Shiwandashan, Guangxi Province, China, and the specimen IEBR.A 4848 of *R.johnsi* from Vietnam. Abbreviation: TSR, transverse skin ridges.

In the *R.johnsi* group, the undescribed species could be identified from its closely related species on morphology. ANOVA tests indicated that on the number of transverse skin ridges on the dorsal surface of thighs and tibias, the undescribed species significantly differs from its closely related species (all *p*-values < 0.01; Table 3; Fig. 3). The undescribed species has larger number of transverse skin ridges either on thighs (mean 14.0 ± 1.7, range 12–16, n = 6), on tibias (mean 12.5 ± 2.0, range 9–15, n = 6), and totally on the two body parts (mean 26.5 ± 2.7, range 22–29, n = 6) than *R.sangzhiensis* (on thighs mean 9.7 ± 1.3, range 7–11, n = 7; on tibias mean 10.1 ± 1.1, range 8–11, n = 7; and totally on the two parts mean 19.9 ± 1.8, range 17–22, n = 7), *R.zhengi* (on thighs mean 10.0 ± 1.8, range 7–15, n = 22; on tibias mean 8.1 ± 1.3, range 6–12, n = 22; and totally on the two parts mean 18.1 ± 2.7, range 15–22, n = 22), *R.johnsi* from Vietnam (on thighs mean 9.3 ± 2.3, range 8–12, n = 3; on tibias mean 9.0 ± 1.0, range 8–10, n = 3; and totally on the two parts mean 18.3 ± 3.2, range 16–22, n = 3), and *R.johnsi* from Guangxi, China (on thighs mean 10.3 ± 0.9, range 9–12, n = 9; on tibias mean 8.8 ± 1.8, range 6–12, n = 9; and totally on the two parts mean 19.1 ± 2.0, range 16–22, n = 9).

**Table 3.** Comparisons on number of skin ridges on thighs and tibias between *Ranawuyiensis* sp. nov. and its closely related species. *P*-value was resulted from One-way ANOVA test. Significant level at 0.05.

	*Ranawuyiensis* sp. nov. (RW)		*R.sangzhiensis* (RS)		*R.zhengi* (RZ)		*R.johnsi* Vietnam (RJV)		*R.johnsi* Guangxi, China (RJC)		*P*-value			
	n = 6		n = 7		n = 25		n = 3		n = 9					
	Range	Mean ± SD	Range	Mean ± SD	Range	Mean ± SD	Range	Mean ± SD	Range	Mean ± SD	RW vs. RS	RW vs. RZ	RW vs. RJV	RW vs. RJC
Number of skin ridges on thighs	12–16	14.0 ± 1.7	7–11	9.7 ± 1.3	7–15	10.0 ± 1.8	8–12	9.3 ± 2.3	9–12	10.3 ± 0.9	0.000	0.000	0.000	0.000
Number of skin ridges on tibias	9–15	12.5 ± 2.0	8–11	10.1 ± 1.1	6–12	8.1 ± 1.3	8–10	9.0 ± 1.0	6–12	8.8 ± 1.8	0.005	0.000	0.001	0.000
Total number of skin ridges on thighs and tibias	22–29	26.5 ± 2.7	17–22	19.9 ± 1.8	15–22	18.1 ± 2.7	16–22	18.3 ± 3.2	16–22	19.1 ± 2.0	0.000	0.000	0.000	0.000

**Table 4.** Morphometric comparisons between the adult male specimens of *Ranawuyiensis* sp. nov. and its closely related species. Units given in mm. See abbreviations for the morphological characters in Materials and methods section. *P*-value was resulted from One-way ANOVA test. Significant level at 0.05. *P*-value < 0.05 denoted as bold.

	*Ranawuyiensis* sp. nov. (RW)		*R.sangzhiensis* (RS)		*R.zhengi* (RZ)		*R.johnsi Vietnam* (RJV)		*R.johnsi* Guangxi, China (RJC)		*P*-value for male			
	n = 4		n = 5		n = 22		n = 2		n = 8					
	Range	Mean ± SD	Range	Mean ± SD	Range	Mean ± SD	Range	Mean ± SD	Range	Mean ± SD	RW vs. RS	RW vs. RZ	RW vs. RJV	RW vs. RJC
SVL	41.4–45.6	42.9 ± 1.9	44.5–51.4	46.9 ± 2.8	37.9–45.7	42 ± 2.0	44.3–47.2	45.7 ± 2.1	40.7–46.2	44.3 ± 1.6	**0.005**	0.457	0.113	0.243
HDL	10.3–14.7	12.7 ± 2.2	14.1–16.6	15.2 ± 1	12.4–15.3	14.2 ± 0.8	14.0–15.6	14.8 ± 1.2	14.0–16.2	15.2 ± 0.7	**0.052**	**0.001**	0.125	**0.001**
HDW	12.3–15	13.5 ± 1.3	14.5–15.2	14.9 ± 0.3	13.1–15.9	14.1 ± 0.7	13.3–14.6	13.9 ± 0.9	13.4–15.9	15 ± 0.8	0.559	**0.004**	0.400	**0.004**
SL	5.7–7.1	6.4 ± 0.7	6.3–7.2	6.6 ± 0.4	5.2–6.2	5.7 ± 0.2	6.3	6.3 ± 0.0	6.2–7.5	7.0 ± 0.4	0.207	**0.016**	0.148	0.109
SNT	2.2–3.2	2.8 ± 0.5	3.2–3.5	3.4 ± 0.1	2.4–3.2	2.8 ± 0.2	3.4–3.8	3.6 ± 0.3	2.8–3.5	3.2 ± 0.3	**0.021**	0.296	**0.003**	**0.014**
IND	4.1–5	4.6 ± 0.4	4.6–5.1	4.8 ± 0.2	3.4–4.9	4.1 ± 0.4	3.5–3.9	3.7 ± 0.3	3.8–4.6	4.2 ± 0.3	0.509	**0.033**	**0.000**	**0.003**
IOD	3.5–4.1	3.8 ± 0.2	3.4–4.2	3.7 ± 0.3	2.6–3.7	3.2 ± 0.3	4.3–4.9	4.6 ± 0.4	3.5–4.3	3.9 ± 0.3	**0.045**	**0.002**	0.159	0.654
UEW	2.5–2.8	2.6 ± 0.1	3.3–3.9	3.5 ± 0.2	2.5–3.6	3.1 ± 0.3	2.7–2.9	2.8 ± 0.1	3.2–4.4	3.7 ± 0.4	**0.000**	**0.000**	0.967	**0.000**
ED	5.0–5.7	5.3 ± 0.3	4.8–5.6	5.2 ± 0.3	4.3–5.7	5.1 ± 0.4	4.5–4.8	4.6 ± 0.2	4.6–5.5	5.1 ± 0.3	**0.005**	0.704	**0.000**	**0.023**
TYD	3.7–4.5	3.9 ± 0.4	3.4–4.4	3.8 ± 0.4	2.9–3.6	3.2 ± 0.2	3.2–4.3	3.7 ± 0.7	3.3–4.3	3.8 ± 0.4	**0.009**	**0.000**	0.061	0.124
LAL	8.9–20.7	14.8 ± 6.6	9.7–10.8	10.3 ± 0.5	8.6–10.6	9.6 ± 0.5	20.4–22.6	21.5 ± 1.5	18.6–22.6	20.8 ± 1.3	**0.000**	**0.000**	**0.002**	**0.000**
HAL	11.4–11.8	11.5 ± 0.2	10.5–12.6	11.6 ± 0.8	10.0–12.4	11.0 ± 0.6	11.3–12.2	11.7 ± 0.6	10.4–14.5	12.1 ± 1.2	**0.044**	0.503	0.396	0.724
LW	3.5–4.4	3.9 ± 0.4	5.1–5.7	5.4 ± 0.2	3.6–5.4	4.4 ± 0.4	4.4–5.0	4.7 ± 0.4	4.7–6.1	5.4 ± 0.4	**0.000**	**0.000**	**0.016**	**0.000**
FIL	4.6–5.3	5.0 ± 0.3	5.0–6.1	5.4 ± 0.4	4.4–5.9	5.1 ± 0.4	5.1–6.6	5.8 ± 1.0	3.9–5.3	4.6 ± 0.5	0.835	0.485	0.259	**0.020**
FIIL	4.0–4.3	4.2 ± 0.1	4.2–4.7	4.4 ± 0.3	3.0–5.0	4.1 ± 0.4	4.8–5.2	5.0 ± 0.2	3.7–5.1	4.3 ± 0.4	0.645	0.921	0.176	0.823
FIIIL	6.8–7.5	7.1 ± 0.3	7.3–8.8	8.1 ± 0.6	6.4–8.2	7.3 ± 0.4	7.7–8.1	7.9 ± 0.3	6.0–7.8	6.8 ± 0.5	0.448	0.231	0.548	**0.021**
FIVL	4.6–4.7	4.6 ± 0.1	4.5–5.6	5.2 ± 0.5	4.0–4.9	4.4 ± 0.3	4.8–5.7	5.2 ± 0.6	4.2–5.4	4.6 ± 0.4	0.741	0.470	0.436	0.399
THL	23.2–26.6	24.8 ± 1.5	23–26.8	24.6 ± 1.5	19.9–26.0	22.9 ± 1.2	24–27.3	25.3 ± 1.4	23–27.3	25.3 ± 1.4	**0.001**	**0.011**	0.884	0.649
TL	26.4–29.3	27.8 ± 1.2	27.1–30.1	28.5 ± 1.3	23.4–27.7	25.4 ± 1.0	26.9–31.2	29.1 ± 3.1	26.6–30.9	28.7 ± 1.5	**0.015**	**0.002**	0.518	0.928
TW	4.3–4.5	4.4 ± 0.1	5.5–6.9	6.3 ± 0.5	4.5–5.9	5.1 ± 0.4	4.9	4.9 ± 0.0	4.5–6.2	5.3 ± 0.6	**0.000**	**0.000**	0.398	**0.001**
TFL	34.8–38	36.7 ± 1.4	35.2–39.5	37.9 ± 1.9	31–37.7	35 ± 1.6	36.0–40.4	38.2 ± 3.1	33.9–40	36.4 ± 2.1	**0.035**	0.202	0.440	0.083
FL	24.1–26.4	25.4 ± 1	24.1–27.1	25.8 ± 1.4	21.2–25.7	23.7 ± 1.1	24.7–27.5	26.1 ± 2.0	23.4–27	25.2 ± 1.4	**0.013**	**0.045**	0.309	0.117

On morphometric characters, the results of One-way ANOVA showed that in male, the undescribed species was significantly different from *R.sangzhiensis* on SVL, HDL, SNT, IOD, UEW, ED, TYD, LAL, HAL, LW, THL, TL, TW, TFL, and FL (all *p*-values < 0.05), significantly different from *R.zhengi* on HDL, HDW, SL, IND, IOD, UEW, TYD, LAL, LW, THL, TL, TW, and FL (all *p*-values < 0.05), significantly different from *R.johnsi* from Vietnam on SVL, SNT, IND, ED, TYD, and LAL (all *p*-values < 0.05), and significantly different from *R.johnsi* from Guangxi, China on HDL, HDW, SNT, IND, UEW, ED, LAL, LW, FIL, FIIIL, and TW (all *p*-values < 0.05; Table 4).

In total, molecular phylogenetic analyses and morphological comparisons indicated that our specimens from Wuyi Mountain, Fujian Province, China should be classified into the *R.johnsi* group, and are significantly divergent from its closely related species. The specimens should represent a new species which is described as following section.

### Taxonomic account

#### Rana wuyiensis sp. nov.

Taxon classificationAnimaliaAnuraRanidae

BF306E1A-EAA9-5E97-9E74-3F497316CEA1

http://zoobank.org/66BA9380-4998-4EAF-9B58-7E0AEAB2C58C

**Figure 4.**
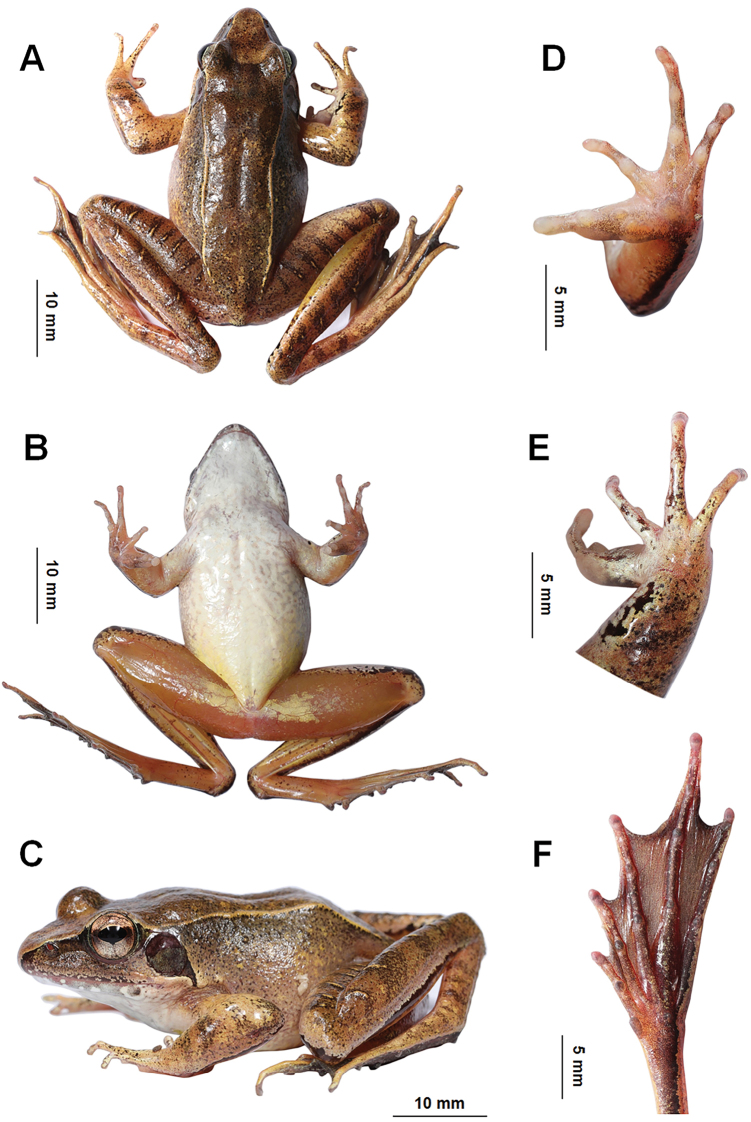
Photos of the holotype CIB WY20200913003 of *Ranawuyiensis* sp. nov. in life **A** dorsal view. **B** ventral view **C** lateral view **D** ventral view of hand **E** dorsal view of hand **F** ventral view of foot.

**Figure 6.**
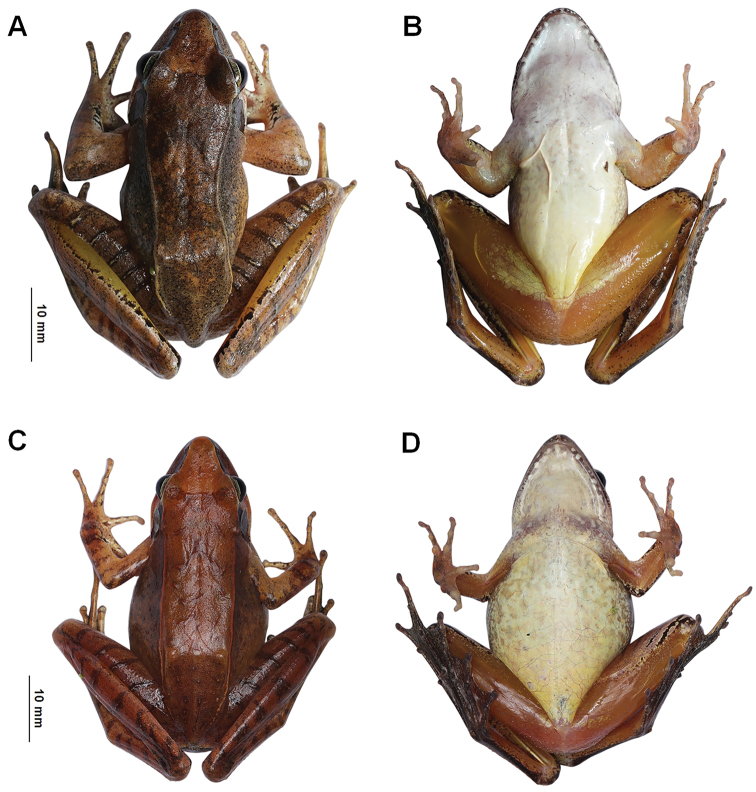
Color variation in *Ranawuyiensis* sp. nov. **A** dorsal view of the adult male specimen CIB WY20200913001 **B** ventral view of CIB WY20200913001 **C** dorsal view of the adult female CIB WY20200913002 **D** ventral view of CIB WY20200913002.

Figs 3 , 4 , 5 , 6 ; Tables 1 , 2 , 3 , 4 , Suppl. material 1

##### Material examined.

***Holotype*** (Figs 4, 5). CIB WY20200913003, adult male, collected by Yanqing Wu on 13 September 2020 from Wuyishan National Park (27.760°N, 117.743°E, ca. 1341 m a.s.l.), Wuyishan City, Fujiang Province, China. ***Paratypes*.** Five adult specimens from the same place as holotype collected by Yanqing Wu. One female CIB WYS20200829001 and two males CIB WYS20200829002 and CIB WY20200829003 were collected on 29 August 2020. One female CIB WY20200913002 and one male CIB WY20200913001 were collected on 13 September 2020.

##### Other material examined.

Six tadpoles collected from the same place as holotype (Table 1) by Yanqing Wu on 01 November 2020.

##### Diagnosis.

*Ranawuyiensis* sp. nov. is distinguished by a combination of the following morphological characters: body size medium, SVL 41.4–45.6 mm (42.9 ± 1.9 mm, n = 4) in adult males, and 47.6–50.3 mm (n = 2) in adult females; lateroventral grooves present on tip of toes; transverse skin ridges distinctly present on the dorsal surface of thighs and tibias, the number large (mean 26.5 ± 2.7, range 22–29, n = 6); adult male with a pair of internal subgular vocal sacs; webbing on fourth toes reaching the tip of toe; breeding males possess creamy white nuptial pad with tiny hoar velvety spines on the dorsal surface of the first finger, divided into three parts.

##### Etymology.

The specific name *wuyiensis* is in reference to the type locality, Wuyi Mountain, Fujian Province, China.

##### Suggested common name.

Wuyi Brown Frog (in English), Wuyi Lin Wa (in Chinese; 武夷林蛙).

##### Description of holotype

**(Figs 4, 5). Adult male**; SVL 41.4 mm. Head significantly longer than wide (HDW/HDL ratio = 0.85); snout pointed and projecting over lower lip; nostril closer to tip of snout than eye; canthus rostralis distinct; internasal distance distinctly wider than interorbital distance (IOD/IND ratio = 0.81); loreal region slightly oblique and concave; upper eyelids narrower than interorbital distance; tympanum rounded, diameter three quarters of eye (TD/ED ratio = 0.75), and separated from eye by a short distance about one quarter of tympanum diameter; tympanic rim feebly elevated; pupil oval and horizontal, notched at middle lower margin; a skin fold present posterior to tympanum, disconnected with dorsolateral fold, swollen near shoulder; vomerine teeth in two short row, four or five for each, oblique and separated by a distance about one row of teeth; tongue deeply notched posteriorly, depth about one sixth of entire tongue length; a pair of internal subgular vocal sacs present, openings slit like, small, length as wide as finger tips, positioned at on inner mandible near the corners of mouth.

Forearms moderate, width 0.09 ratio of SVL; hand 0.27 ratio of SVL; fingers elongated, with narrow lateral fringes, rudimentary webbed, webbing formula I 3⅔ – 2⅔ II 2½ – 3½ III 3½– 3 IV; tips of fingers rounded, not swollen, without lateroventral groove; finger II distinctly shorter than I, relative finger lengths II < I < IV < III; subarticular tubercles prominent, rounded; supernumerary tubercles indistinct, oval, present on bases of all fingers; inner metacarpal tubercle distinct, near oval, positioned near inner surface of base of finger I, inner side partially covered with nuptial pad; two outer metacarpal tubercles partially separated near the joint of metacarpals of fingers III and IV, the inner oval and larger, the outer elongated and smaller; nuptial pad present on inner and dorsal surface of finger I, covered with velvety spines, partially divided into three parts, the basal part on inner side of inner metacarpal tubercle, the middle part largest, on third phalanx, the distal part smallest, on first and second phalanxes.

Hindlimbs long, tibia 0.64 ratio of SVL and length of foot and tarsus 0.84 ratio of SVL; thigh shorter than tibia, heels overlap when hindlimbs flexed at right angles to axis of body; tibio-tarsal articulation reaching far beyond snout when hindlimb stretched forward along body; toes entirely webbed, inner edge of toe I and outer edge of toe V with narrow lateral fringe, relative toe lengths I < II < III < V < IV, toes webbing formula: 1⅓ – 2 II 1⅓ – 2⅓ III 1½ – 2⅔ IV 3 – 1⅓ V; tip of toes somewhat flat, lateroventral grooves present on all tip of toes and disconnected at middle of front edge; subarticular tubercles prominent and oval; supernumerary tubercles absent; inner metatarsal tubercle oval and prominent, outer metatarsal tubercle rounded, indistinct.

Dorsal skin smooth, supratympanic fold absent; dorsolateral folds distinct, narrow, extending from edge of upper eyelid to hip, not curve above tympanum. Ventral skin smooth, skins around cloaca with numerous flat tubercles. Skin on hindlimbs with transvers paralleled ridges, eight on both thighs, six and seven on left and right tibias, four and two on left and right tarsal. Tarsal fold present.

##### Coloration in life

**(Fig. 4).** Dorsal surface basically medium brown, scattered with dense dark brown pigments all over; dorsolateral skin folds and skin ridges on dorsal limbs yellow brown with deep drown fringes; five ambiguous deep brown cross bands present on dorsal forelimbs; irregular black patches present on inner surface of forearm near wrist, anterior knee and lateral tibia; lower edge of canthus rostralis dark brown; skins on tympanum and anterior to the fold behind tympanum deep brown; ventral skin basically cream white on body and arm; lips light brown with cream white patches; throat, chest, and upper abdomen with irregular light orangish short bars; ventral hindlimbs mostly flesh colored, with a small region near base of tinged yellowish white; ventral hand flesh-colored with brown pigments; ventral feet covered with dense brown pigments. Nuptial pad hoar. Iris mostly copper with dark cracks, regions anterior and posterior to pupil deeper.

##### Coloration in preserve

**(Fig. 5).** Body coloration lighter than in life, dark brown pigments more prominent. Skins between upper eyelids with an ambiguous brown pattern. Ventral body mostly white, with brown pattern; ventral limbs yellowish. Ventral hand and feet greyish. Skins on temporal region with prominent dark patches. Lateral head before eyes blackish. Iris dark with metal luster.

##### Secondary sexual characters.

Breeding males with nuptial pad on dorsal surface of finger I, covered with velvety spines, divided into three parts. Male with a pair of internal subgular vocal sacs.

##### Variations.

For measurements of type series specimens see Tables 4, Suppl. material 1. Coloration of the two females lighter (Fig. 6A), basically yellowish brown. Black edges of dorsolateral fold absent on CIB WY20200913002 (Fig. 6C) and indistinct on CIB WYS20200829001. The number of skin ridges on dorsal thigh range from five to eight. The skin ridges on tibia range from four to eight.

##### Distribution and ecology.

**Figure 7.**
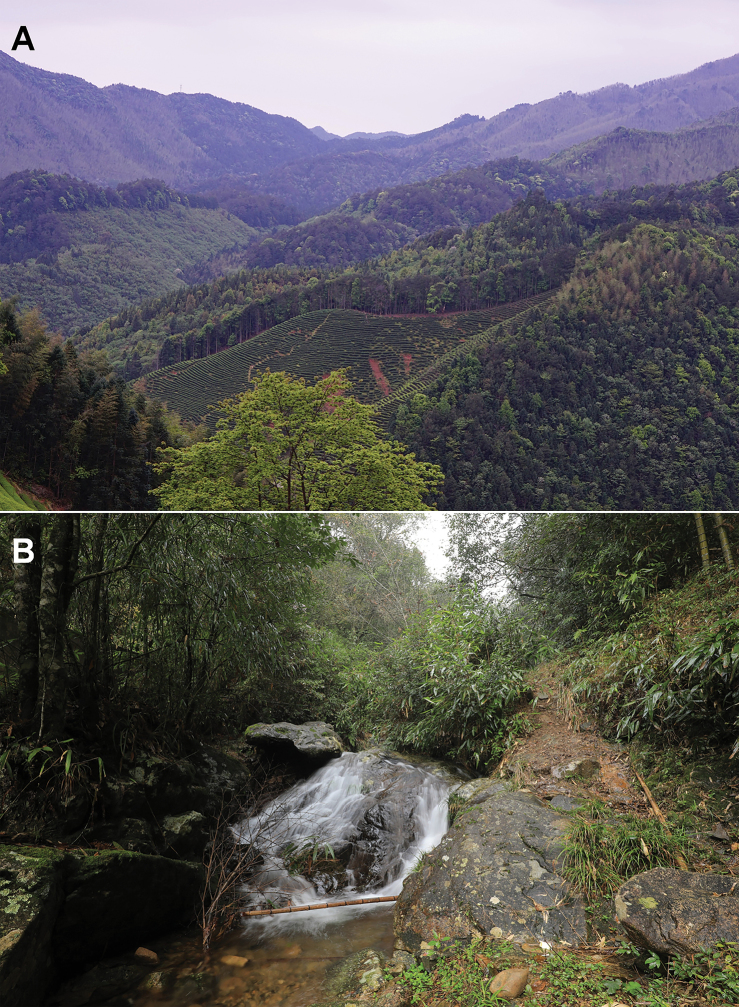
Habitats of *Ranawuyiensis* sp. nov. in the type locality, Wuyi Mountain, Fujian Province, China **A** landscape of montane forests in the type locality **B** a mountain stream in the type locality.

Currently, *Ranawuyiensis* sp. nov. is known from Wuyishan National Park, Wuyishan City, Fujian Province, China. In our surveys from 2017 to 2021, the species was found only at one site. All individuals of the new species used in this work were collected from a stream and nearby grassland under the evergreen broad-leaf forest (Fig. 7). Six adult individuals and some very small tadpoles at early stages of development were found in the late August and early September. Only relative larger and middle-staged tadpoles were collected in the early November. This suggests that the breeding season of this species may begin in July or early August.

## Discussion

Our results based on mitochondrial DNA and nuclear DNA of several populations of *R.zhengi* and *R.sangzhiensis* indicated that the two groups have very low genetic divergence. This is identical to the results of previous molecular phylogenetic analyses in Wan et al. (2020). In addition, we did not find morphological characters for separating the two groups, being consistent with the results of Jiang et al. (1997) and Fei et al. (2009). Based on this evidence, we support the proposal that *R.zhengi* should be synonymized with *R.sangzhiensis*. Accordingly, *R.sangzhiensis* is at least distributed from southwestern part of Sichuan to western Hunan provinces, China. On the contrary, *Ranawuyiensis* sp. nov. differs from its closely related species not only on morphology but also on molecular data, supporting the separation of the new species.

Moreover, the divergence between *Ranawuyiensis* sp. nov. and its closely related species in the *R.johnsi* group is likely corresponding to their separated distributional ranges (Fig. 1). Wuyi Mountain is located at the southeastern edge of the mainland China, far from the “west” distributional ranges of *R.johnsi* and *R.sangzhiensis* in southwestern China (at least > 400 km in a straight line between them; Fig. 1), and the distribution ranges belong to different biota (e.g., Zhang 2009; Fei et al. 2010). This indicates that vicariance might be the primary factor for the speciation of the species. Whatever, the discovery of the new species greatly expanded the distributional range of the *R.johnsi* group to the southeastern China and would promote exploring the biogeographical patterns in the frog group.

However, to date, *Ranawuyiensis* sp. nov. was found only at one site in Wuyi Mountain, and it probably has a low population size according to our eleven-times surveys which included forty sites every time in April, June, and August from 2018 to 2021. Although this site is in the central part of the Wuyishan National Park, the breeding habitat is vulnerable due to local human activities especially tea plantation (Fig. 7A) and/or local nature disaster (for example, the novel rainstorm in 2020 in Wuyi Mountain; our unpublished data). Therefore, we need to understand its population status and major threats, and then take appropriate actions to prepare strategies for its conservation.

## Supplementary Material

XML Treatment for Rana wuyiensis

## Supplementary materials

Supplementary material 1 Table S1 Data type morphological measurements of adults Explanation note Measurements of adult specimens of *Ranawuyiensis* sp. nov. and its closely related species. Units given in mm. See abbreviations for the morphological characters in Materials and methods section. https://binary.pensoft.net/file/603487This dataset is made available under the Open Database License (http://opendatacommons.org/licenses/odbl/1.0/). The Open Database License (ODbL) is a license agreement intended to allow users to freely share, modify, and use this Dataset while maintaining this same freedom for others, provided that the original source and author(s) are credited.Bin Wang
